# Social Behaviours under Anaerobic Conditions in *Pseudomonas aeruginosa*

**DOI:** 10.1155/2012/405191

**Published:** 2012-02-09

**Authors:** Masanori Toyofuku, Hiroo Uchiyama, Nobuhiko Nomura

**Affiliations:** Graduate School of Life and Environmental Sciences, University of Tsukuba, Tsukuba, Ibaraki 305-8572, Japan

## Abstract

*Pseudomonas aeruginosa* is well adapted to grow in anaerobic environments in the presence of nitrogen oxides by generating energy through denitrification. Environmental cues, such as oxygen and nitrogen oxide concentrations, are important in regulating the gene expression involved in this process. Recent data indicate that *P. aeruginosa* also employs cell-to-cell communication signals to control the denitrifying activity. The regulation of denitrification by these signalling molecules may control nitric oxide production. Nitric oxide, in turn, functions as a signalling molecule by activating certain regulatory proteins. Moreover, under denitrifying conditions, drastic changes in cell physiology and cell morphology are induced that significantly impact group behaviours, such as biofilm formation.

## 1. Introduction

It is well acknowledged that bacteria exhibit social behaviours by communicating with each other through signalling molecules or by developing a community known as biofilm. The social behaviour of bacteria is of great interest to researchers, and *Pseudomonas aeruginosa* is one of the most studied bacterial model organisms.

*P. aeruginosa* has a flexible metabolism that can utilise nitric oxides as alternative electron acceptors to produce energy when oxygen is depleted [1]. This process is called denitrification and is also performed by many other bacteria. The stepwise process of denitrification in *P. aeruginosa* is as follows: NO_3_^−^ → NO_2_^−^ → NO → N_2_O → N_2_. The sequential steps are catalysed by the enzymes NO_3_^−^ reductase (NAR), NO_2_^−^ reductase (NIR), NO reductase (NOR), and N_2_O reductase (N_2_OR), respectively [2]. This process is important in the nitrogen cycle to produce nitrogen gases from NO_3_^−^ and NO_2_^−^. Moreover, recent studies indicate that the denitrification process is related to the virulence of this bacterial species. *P. aeruginosa* is notorious as an opportunistic pathogen that infects immunocompromised patients, such as cystic fibrosis (CF) patients. How the bacteria adapt to the host environment is important in terms of its pathogenesis. The CF airway has been described as a microaerobic to anaerobic environment [3, 4]. Independent studies indicate the expression of denitrifying genes in the CF lung, suggesting that denitrification is important for the pathogenicity of *P. aeruginosa* [5, 6]. Thus, an understanding of the physiology under anaerobic conditions is important for the understanding of bacterial virulence under such conditions.

 While there are many excellent reviews available about the social behaviours of *P. aeruginosa* under aerobic conditions, few have focused on anaerobic conditions. In this paper, we examine the social behaviour of *P. aeruginosa* under anaerobic conditions.

## 2. Denitrification Regulation by Physicochemical Conditions

The expression of denitrifying enzymes is controlled by a sophisticated regulatory network that responds to low oxygen conditions and the availability of nitrate or nitrite. The master regulator that monitors oxygen concentration is the ANR (anaerobic regulation of arginine deiminase and nitrate reduction) regulatory protein [7]. The active form of ANR contains an [4Fe-4S]^2+^ cluster that is destroyed in the presence of oxygen [8]. ANR induces genes that are involved in producing energy under low-oxygen conditions or anaerobic conditions. One of these genes, the *cbb_3_*-2 terminal oxidase, has a high affinity for oxygen, indicating that it plays a role under low-oxygen conditions [9, 10]. Other genes induced by ANR include the genes involved in fermentation [11]. In addition to these genes, ANR induces other transcriptional regulators involved in denitrification, NarXL, and DNR (dissimilative nitrate respiration regulator) [12, 13]. NarX and NarL comprise a two-component regulatory system that responds to nitrate. The sensor kinase NarX detects nitrate and activates the response regulator NarL, which regulates the transcription of *narK1*, *nirQ*, and* dnr* [12]. In addition, NarL partially represses the expression of arginine fermentation genes, enabling the bacteria to benefit from the more energetically efficient denitrification instead of low energy-yielding fermentation in the presence of nitrate under anaerobic conditions [14]. DNR is activated by binding to NO, and the active DNR regulator activates transcription of all four denitrifying reductases [12, 13, 15].

## 3. Cell-Cell Communication Signals in *P. aeruginosa*

In* P. aeruginosa*, two chemically distinct types of signalling molecules have been characterised in detail. One type consists of the *N*-acyl-_L_-homoserine lactone (AHL) signals. AHLs are produced widely in gram-negative bacteria, and *P. aeruginosa* is known to possess two AHL signaling systems, the LasR-LasI (*las*) and the RhlR-RhlI (*rhl*) systems [16]. LasI directs the synthesis of the AHL signal *N*-(3-oxododecanoyl)-_L_-homoserine lactone (3-oxo-C_12_-HSL) [17, 18], and RhlI directs the synthesis of another AHL signal, *N*-butyryl-_L_-homoserine lactone (C_4_-HSL) [19, 20]. These AHL signals have cognate receptors (LuxR proteins), LasR [21] and RhlR [22], that are activated by 3-oxo-C_12_-HSL and C_4_-HSL, respectively. In addition to the LasR and RhlR receptors, two additional LuxR homologues, QscR and VqsR, which are orphan LuxR proteins and not associated with a cognate AHL synthase, have been found. QscR binds to a variety of AHL molecules with different side chains, while the AHL molecule that binds to VqsR is unknown [23, 24]. The other type of signalling molecules in *P. aeruginosa* include 2-alkyl-4-quinolone (AQ). The AQ signalling molecules are produced by the product of the *pqsABCDE *operon together with the product of the *pqsH* gene and converts 2-heptyl-4-quinolone (HHQ) to the *Pseudomonas* quinolone molecule (PQS; 2-heptyl-3-hydroxy-4-quinolone) [25]. HHQ is also produced in bacteria other than *P. aeruginosa* [26]. In* P. aeruginosa*, both PQS and HHQ are able to regulate the *pqsABSDE *operon via a transcriptional regulator, PqsR (MvfR) [27]. Interestingly, PQS is carried by outer membrane vesicles (OMVs), which are thought to target neighbouring cells [28, 29].

## 4. Denitrification Regulation by Signalling Molecules

**Figure 1:**
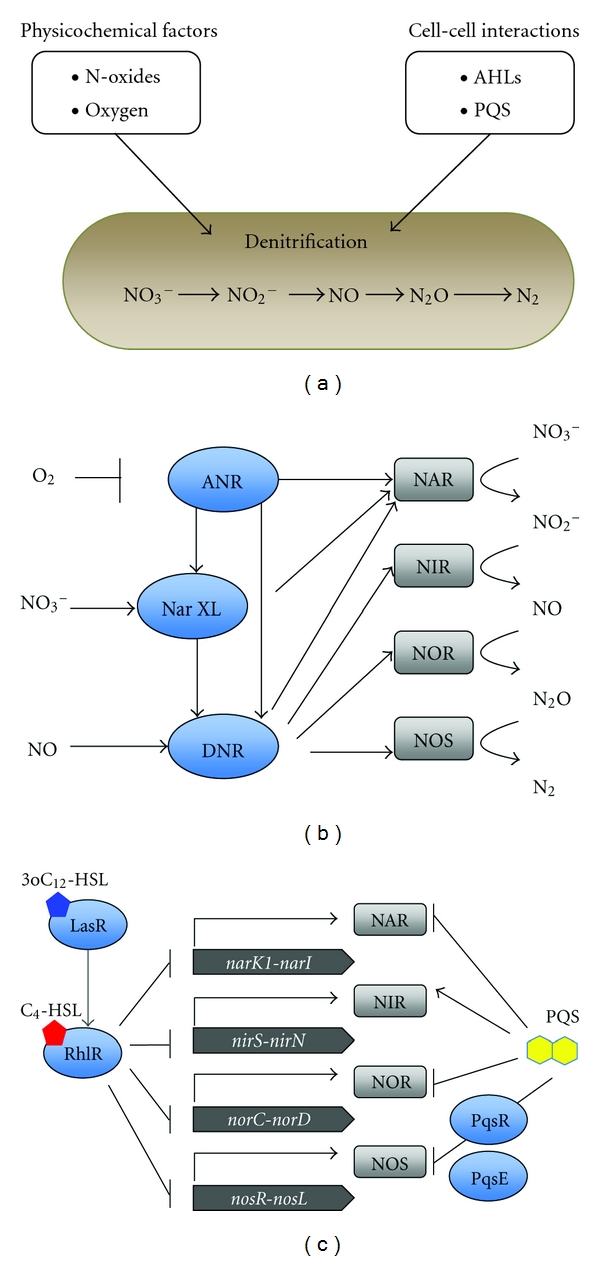
(a) Denitrification regulation in *P. aeruginosa*. Denitrification is regulated by physiochemical conditions, such as oxygen concentration and the availability of nitric oxides as well as cell-cell communication molecules. (b) Denitrification regulation by physiochemical factors in *P. aeruginosa.* ANR is activated under low-oxygen tension conditions, which increases the transcription of the NarXL two-component system and DNR. NarXL responds to nitrate and activates the expression of DNR together with ANR. DNR is activated in the presence of NO and induces the transcription of the four reductases. The *narK1K2GHJI *operon, which encodes nitrate/nitrite transporters and the structural gene for NAR, is also induced by ANR and NarL. (c) Denitrification regulation by cell-cell communication. C_4_-HSL and 3-o-C_12_-HSL repress the transcription of denitrifying reductases via their cognate receptors RhlR and LasR. Regulation by LasR is dependent on RhlR. PQS represses the activity of the NAR, NOR, and NOS enzymes while activating the NIR enzyme. PqsR and PqsE are involved in the PQS effect on NOR, while the effect on the other three enzymes does not require PqsR or PqsE.

As mentioned above, denitrification is well regulated by the physiochemical environment. In addition, denitrification is regulated by cell-cell communication molecules. Interestingly, all three cell-cell signalling molecules, C_4_-HSL, 3-oxo-C_12_-HSL, and PQS, repress denitrification. AHLs and PQS affect denitrification in different manners. In the study conducted by Yoon et al. [30], it was demonstrated that the activity of the denitrifying enzymes is higher in *rhlR* mutants compared to its parent strain. A microarray study suggested that the denitrifying genes are regulated by AHLs [31]. Following these observations, it was demonstrated in detail that, indeed, AHLs regulate denitrifying activity. Both C_4_-HSL and 3-oxo-C_12_-HSL repressed denitrifying activity via their cognate regulator, RhlR or LasR, by regulating the expression of the denitrifying genes [32]. Regulation by the *las *quorum-sensing system was dependent on the *rhl *quorum-sensing system, suggesting hierarchical regulation by the *las *system over the *rhl *system in denitrification regulation, although the precise mechanism of denitrification regulation by AHLs has yet to be identified. In *P. aeruginosa* isolates from CF patients, mutations in the *lasR *gene are often found [33, 34]. Considering the fact that there are microenvironments with low oxygen tension inside the CF lung, it is possible that the *lasR* mutation confers a growth advantage by the activation of denitrification Figure 1 [35].

 While the AHLs regulate the transcription of denitrifying genes, PQS affects the activity of denitrifying enzymes posttranscriptionally. It has been shown that NAR and NOR activity is repressed and NIR activity is increased in the presence of PQS [36]. Furthermore, when the PQS molecule was added to a crude extract containing denitrifying enzymes, NO_3_^−^-respiration activity and NOR activity were repressed [36]. This was the first study to demonstrate that a signalling molecule affects enzyme activity in a direct manner. The transcription of denitrifying genes was unaffected by PQS under anaerobic conditions [36], while microarray studies suggested that PQS represses *nar* gene transcription under aerobic conditions [37], indicating that there may be several pathways in denitrification regulation by PQS. In addition to working as a signalling molecule that activates its cognate receptor, the PQS molecule is shown to have multiple functions, such as chelating iron, balancing the production of reacting oxygen species, and inducing outer membrane production [28, 38–40]. Iron concentration is a key environmental condition for the PQS effect on denitrification because excess amounts of iron inhibit this effect [36]. Interestingly, PQS affects the aerobic and anaerobic growth of bacterial species other than *P. aeruginosa*, indicating its broad impact on the bacterial community [41].

## 5. Cell-Cell Signalling under Denitrifying Conditions

While cell-cell communication has a wide impact on cell physiology, most of the studies in *P. aeruginosa* have been performed under aerobic conditions, and there have been only a limited number of studies concerning the impact of the cell-cell communication systems under anaerobic conditions. Expression of *lasR*,* lasI*,* rhlR*, and *rhlI* was shown to be altered under denitrifying conditions [31, 42]. Furthermore, a recent study measuring the AHLs under denitrifying conditions revealed that the production of AHLs is significantly lower compared to aerobic cultures [43]. The exact mechanism of this attenuation in signal production has not been revealed, but it is proposed that the limitation of the acyl carrier proteins leads to the low level of signalling molecules [43]. Interestingly, the AHL signalling systems under denitrifying conditions still actively regulate genes to some extent, as has been demonstrated by the regulation of denitrifying activity using AHL production-defective mutant strains [30, 32]

PQS production is also suppressed under anaerobic conditions [36, 44]. In this case, the enzymes that convert HHQ to PQS require oxygen. Therefore, under anaerobic conditions, hydroxylation of HHQ does not occur, and thus, PQS synthesis is prevented [44]. However, under these conditions, a sufficient amount of HHQ that can induce *pqsABCDE* transcription is present [44]. It was shown that both PQS and HHQ bind to the PqsR transcriptional regulator, although PQS is approximately 100-fold more potent in stimulating the activation of PqsR [27]. These results imply that HHQ may play an important role in cell-cell communication under denitrifying conditions. In fact, HHQ is used as a signalling molecule in several other bacteria [26], but the impact of HHQ as a signalling molecule has yet to be fully understood in *P. aeruginosa*.

 Although production of all three signalling molecules is attenuated under anaerobic conditions, the exogenous addition of these signalling molecules restore the transcription of target genes [36, 43]. These results indicate that the cells under denitrifying conditions are altered in producing signalling molecules, but they are still able to respond to them. It is important to consider that the natural habitat of bacteria is not a stable environment and conditions, such as oxygen concentration, are likely to fluctuate. Moreover, even under the same environmental conditions, oxygen-limited patches are produced by the cells itself as observed in biofilms [45]. In these cases, it is possible that the signalling molecules produced in one growth condition affect the cells in other growth conditions. One example is the PQS effect on denitrification. As mentioned above, PQS is not produced at a sufficient amount under anaerobic conditions [36, 44]; however, when bacteria were grown under oxygen-limiting conditions in which oxygen was depleted depending on cell growth, denitrifying activity was higher in the non-PQS-producing mutants [36]. This result demonstrates that a signalling molecule produced in one environment can affect the cells in another environment. Because it is known that cell-cell signalling is modulated by environmental factors [46], it would also be interesting to further investigate whether there are any differences in the regulon regulated by the same molecules under aerobic and anaerobic conditions.

## 6. NO Signalling in*  P. aeruginosa*

NO has been studied extensively as a signalling molecule in eukaryotic cells that plays important roles in many biological processes. However, the role of NO in bacteria has not been fully understood. Some recent studies have demonstrated that NO is produced through the oxidation of _L_-arginine in certain gram-positive bacteria [47, 48], as it is in mammals, and it can protect the bacteria from reactive oxygen species. During denitrification, NO is produced as an intermediate by the reduction of nitrite, a process catalysed by the NIR enzyme. In *P. aeruginosa*, the denitrifying pathway is the only biological pathway known to produce NO. This implies that under denitrifying conditions, NO may become a signal that, when produced by one cell, can affect the other cell. A number of regulators that respond to NO have been revealed. As explained above, NO is an important signal for inducing the denitrifying genes. In this case, NO is recognised by the regulatory protein DNR, which regulates the denitrifying genes [15, 49]. The activated DNR recognises the conserved DNA binding site (ANR box) in promoters to regulate transcription. A recent comprehensive study to determine ANR and DNR regulons suggested that, in addition to the denitrifying genes, the transcription of three genes, C4-dicarboxylate transport (PA1183), a hypothetical protein (PA3519), and the RND-type efflux pump *mexG* (PA4205), is influenced by DNR, although no ANR box has been found in the promoter regions of these genes [50]. Still, the roles of these genes under denitrifying conditions or in NO response are not known. Another type of RND efflux pump (*mexEF-oprN*) was suggested to be induced by NO [51]. In this study, the MexT regulator was required for *mexEF-oprN* induction by NO, while the mechanism is still obscure. Nevertheless, it is becoming evident that the expression of efflux pumps is involved in diverse cellular activities that affect the expression of several genes. A recent study has demonstrated that the expression of the MexEF-oprN efflux pump downregulates several genes [52]; thus, NO may impact the expression of these genes through the expression of this efflux pump.

 FhpR is another NO-responsive regulator that, under aerobic conditions, regulates the flavohaemoglobin (*fhp)* gene, the product of which oxidises NO. FhpR is an orthologue of NorR found in *E.coli* and belongs to the *σ* ^54^-dependent family of transcriptional activators [53].

 While high amounts of NO induce genes for NO detoxification [54] and the DNR and FhpR regulatory proteins are likely to regulate these genes, a series of studies have revealed that NO in nontoxic levels regulates the social behaviour of bacteria, such as the dispersal of *P. aeruginosa* in biofilms [55]. The underlying mechanism is not yet fully understood, but a secondary messenger (c-di-GMP) is involved in this process. Low levels of c-di-GMP induce bacterial motility in *P. aeruginosa*, which in turn induce dispersal in biofilms. NO has been shown to increase the activity of enzymes that degrade c-di-GMP. This process requires the chemotaxis transducer BdlA [56]. These data indicate that there is a biological pathway independent of the toxic response that responds to NO.

 Although it is not fully characterised, another NO-responsive gene that regulates biofilm formation has been suggested. This gene product (PA2663) increases biofilm formation by inducing the production of psl exopolysaccharides while reducing swarming and swimming motility [57]. It also increases the production of virulence-related factors, such as pyoverdine, PQS, and elastase. The gene is located within the same operon as the *fhp* gene, indicating that NO will induce its expression. Interestingly, PA2663 has two transmembrane regions and may sense NO, although further study is needed.

 NO has also been suggested to induce virulence factors. A *nirS* mutant deficient in NO production was shown to have a reduced amount of type III secretion system-dependent exotoxin production compared to wild type. This NO-dependent exotoxin induction was confirmed by the addition of exogenous NO donors [58].

 One of the important factors that determines the cellular response to NO is the concentration of NO. High levels of NO are toxic to the cell, but low levels of NO could function as a signal. How NO concentration is modulated in the cell during denitrification is not fully understood, but one possibility is denitrification control by the AHL and PQS molecules because they affect NIR and NOR activity to a different extent. For example, rhl quorum-sensing mutants increase NO production due to the imbalance of NIR and NOR activity, and this induces cell death under denitrifying conditions [30]. PQS upregulates NO-producing NIR enzyme activity, while it downregulates the NO-reducing NOR enzyme activity, suggesting that PQS would induce NO accumulation [36].

## 7. Biofilm Formation under Denitrifying Conditions

A study by Yawata et al. [59] showed that filamentous cells emerge extensively in biofilms formed under denitrifying conditions. This was followed by the study by Yoon et al., which indicated that the filamentation is due to a defect in cell division, and filamentation of the cells is correlated with biofilm formation [60]. It was also suggested in this study that the filamentation is induced by NO [60]. Filamentation of cells has long been characterised as a trait of cell morphology under stressful conditions, such as UV light exposure, antibiotic treatment, and nutrient deprivation. In contrast, it is known that filamentation of the cell is a part of cell differentiation in bacteria, such as *Proteus mirabilis *[61]. Currently, it is not known whether the filamentation in denitrifying biofilms is a response to NO toxicity or an adaptive response to denitrifying conditions or whether there is a specific signalling pathway that initialises cell filamentation in *P. aeruginosa*. Nevertheless, morphological changes may impact cell physiology and behaviours and provide advantages for survival under certain circumstances [62].

 Most of the studies investigating biofilm formation in *P. aeruginosa* have been performed under aerobic conditions. The morphological change in cells under denitrifying conditions causes us to question how the biofilm development process differs under aerobic conditions. Studies using a selected number of mutants have indicated that the same gene may have different effects on biofilm formation under aerobic and anaerobic conditions [30]. These studies suggest the possibility that the biofilm development procedure differs under denitrifying conditions.

## 8. Perspectives

As our knowledge of energy metabolism under anaerobic conditions expands, new questions arise with respect to the physiology of the cells under those conditions. Under denitrifying conditions, the cells undergo morphological changes [59, 60], and cell physiology is likely to change dynamically with the shift in respiration systems [42]. Understanding the physiology under denitrifying conditions in *P. aeruignosa* is also important from clinical perspectives because anaerobiosis leads to the alteration in antibiotic tolerance, the mechanism of which is not fully understood [45]. Attenuation of the AHL and PQS signalling systems and different biofilm development processes raise further questions about the social behaviours of the bacteria under anaerobic conditions. It will be interesting to investigate whether additional signals or systems that coordinate group behaviours under anaerobic conditions may exist.
